# Role of CA-125 Level as a Marker in the Management of Severe Pre-Eclampsia

**DOI:** 10.3390/healthcare10122474

**Published:** 2022-12-07

**Authors:** Oana Balint, Cristina Secosan, Laurențiu Pirtea

**Affiliations:** 1 Department of Obstetrics and Gynecology, University of Medicine and Pharmacy “Victor Babes”, 300041 Timisoara, Romania; 2 Emergency Clinical City Hospital Timisoara, Obstetrics-Gynecology Clinic, 300231 Timisoara, Romania

**Keywords:** CA-125, pregnancy-induced hypertension, pre-eclampsia

## Abstract

Background and Objectives: Hypertensive disorders of pregnancy remain one of the leading causes of morbidity and mortality in maternal–fetal medicine worldwide, particularly in low-resource settings. Despite extensive research in the last decades, pre-eclampsia prediction and, thus, effective prevention remains an unsolved problem. Current evidence suggests that CA-125, an already recognised tumoral marker and, lately, a valuable severity marker of heart failure, can be used to evaluate pre-eclampsia severity and thus improve the identification and management of high-risk patients; Materials and Methods: This is a case–control study involving 100 pregnant patients over 25 weeks of gestation, grouped based on the severity of hypertension in gestational hypertension (n = 22), non-severe pre-eclampsia (n = 11), severe pre-eclampsia (n = 17), and a control group (normotensive) (n = 50). Clinical and biochemical parameters recommended by the international guidelines for evaluating hypertensive pregnant patients were gathered from every patient in addition to CA-125 levels. The correlation was analysed. Results: Mean CA-125 levels increased with the severity of hypertension from a mean of 8.97 U/mL (±2.84) in the normotensive group to a mean of 21.23 U/mL (±11.18) in the severe pre-eclampsia group. Significant differences were observed between each group. The correlation of CA-125 levels with the assessed clinical and biochemical parameters showed positive correlations with MAP, 24 h proteinuria, and LDH values and negative correlations with platelet count, gestational age at birth, and birth weight Conclusions: The reported results support this marker’s promising role as a severity marker and its potential to improve pre-eclampsia management allowing a better selection of high-risk patients, aiding in decision making related to hospitalisation and/or timing of birth. Further studies are needed to improve the accuracy of the obtained results, identify an accurate cut-off and an optimal time of measurement, and achieve standardisation in measuring the marker.

## 1. Introduction

Hypertensive disorders of pregnancy (HDP) remain one of the biggest problems in public health and maternal–fetal medicine. Current evidence suggests that HDP develops as a two-stage process, with initial deficient placentation followed by dysfunction of the endothelial cells that leads to a systemic inflammatory response and thus a clinical syndrome with various manifestations rather than an individual entity, as it was thought for a long time [1]. HDP includes four major subtypes: gestational hypertension, pre-eclampsia, chronic hypertension, and preeclampsia, complicating chronic hypertension [2]. The complexity and lack of consensus on the definition of each subtype affect the accuracy of reported incidences, with ranges large as 1.8% to 4.4% for gestational hypertension and from 0.2% to 9.2% for pre-eclampsia [3].

Maternal mortality remains significant, particularly in less-developed countries, accounting for 18% of all maternal deaths worldwide [4]. HDP also impacts fetal and neonatal morbidity and mortality, being associated with a 13% risk of perinatal death, a 20% lower birth weight, and a higher overall complication rate compared to a newborn from a normotensive mother [5].

Pre-eclampsia is traditionally defined as systolic blood pressure ≥ 140 mmHg and/or diastolic blood pressure ≥ 90 mmHg, associated with de novo proteinuria above a threshold of 300 mg/24 h or above a urinary protein/urine creatinine ratio ≥ 0.3 mg/mg. This subtype of HDP has been associated with an increased risk for adverse maternal and fetal outcomes compared with other subtypes [6].

Due to the lack of specific effective treatments for HDP, researchers have focused on its prediction and prevention, allowing for early diagnosis and/or better management. Several screening methods have been proposed, including biomarkers, maternal characteristics, Doppler ultrasound, or a combination of different markers [7].

CA-125, or MUC 16, is a large transmembrane glycoprotein part of the mucin family, the subclass of membrane-associated mucins (MAMs). They are glycosylated in specific manners and exhibit functions dependent on the tissue in which they are located [8]. In the epithelial tissues, MAMs contribute to protection against infection and tissue damage [9]. The continuous need to maintain this increased protection against various aggressors implies the need for an intense cellular turnover resulting from a dynamic and balanced process between the biosynthesis, secretion, and degradation of mucins on cell surfaces [10]. Numerous pathological processes cause alterations in the form and/or quantity of secreted mucins. Among them, the most important known pathogenic involvement is in cancer development, with the over-expression and glycosylation aberrations of mucins being reported in many adenocarcinomas [8]. CA-125 has been one of the most used tumoral markers in the last decades, particularly in managing ovarian cancers [11].

CA-125 has been suggested as a possible valuable parameter in predicting preeclampsia evolution after an accidental observation during a study on severe heart failure patients undergoing a heart transplant. Their CA-125 levels (cancer high-risk patients due to their immunosuppressive treatment) decreased significantly after transplantation once their overall cardiac function improved [12]. Subsequent studies provided further evidence supporting this association [13,14]. Currently, two theories bring evidence. The first suggests a possible role in preeclampsia through the immunohistochemically proven presence of this marker in fetal tissues (pleura, peritoneum, and pericardium) and decidual tissue [15,16]. The dynamics of serum CA-125 levels in pregnancy have been studied and are known, with increased values reported in the first trimester and immediately after birth, respectively, suggesting as a potential source the intense processes at the decidual level associated with those periods [17,18,19]. The second theory, supported by recent studies, is based on the observed similarity between the dynamics of CA-125, with significant potential proven as a predictive factor in heart failure, and the dynamics of natriuretic peptide, whose involvement in the pathogenesis of preeclampsia has been investigated in several studies [20,21].

A frequent clinical situation in pre-eclampsia patient management involves a decision regarding hospitalization or out-patient surveillance or establishing the time of birth, especially in extremely preterm patients (less than 28 weeks) and very preterm patients (28 to 32 weeks) where the mother’s well-being has to be balanced with the risk of severe morbidity of the neonate. Those decisions require a thorough evaluation and, ideally, an accurate estimation of the possible evolution of the pre-eclamptic patient. Current routine evaluation parameters of pre-eclamptic patients, like 24 h proteinuria or uric acid, show a poor correlation in predicting maternal and fetal complications in women with pre-eclampsia, thus leading to unnecessary aggressive management for some patients or suboptimal management for others [22,23].

Identifying a marker with a good predictive value for the severity of evolution would allow for better management of patients with pre-eclampsia and provide another criterion in decision-making, especially where currently used parameters do not allow for a clear image of the patient’s status. Moreover, a simple and affordable investigation, such as CA-125, would allow better management of pre-eclampsia in limited resource settings.

This study was designed to analyze the association of CA-125 values an affordable and widely available investigation, with other clinical and biological parameters of known importance in pre-eclampsia severity assessment and its correlation with maternal and fetal outcomes and establish its potential role in the evaluation of pre-eclampsia severity and in pre-eclampsia management.

## 2. Materials and Methods

This case–control study was conducted at a tertiary hospital, Clinical Emergency City Hospital Timișoara, in the Obstetrics-Gynecology Clinic between January 2022 and June 2022. The study population was enrolled from pregnant women attending antenatal care in our hospital. Inclusion criteria were pregnant patients over 25 weeks of gestation, able to attend complete follow-up, and able to provide required data from the first trimester. Exclusion criteria were refusal of enrollment, inability to attend follow-up, a history of chronic hypertension, renal or hepatic disease, diabetes mellitus, known gynecologic pathology (ovarian or uterine disease), multiple pregnancies, known fetal anomalies and pregnancies achieved by ART.

In the proposed period for the study, one hundred pregnant women were ultimately selected and grouped into two main subgroups: 50 pregnant women affected by pregnancy-induced hypertension and 50 normotensive pregnant women. The hypertensive pregnant women subgroup was further devised based on the severity of hypertension in gestational hypertension (*n* = 22), non-severe pre-eclampsia (*n* = 11), and severe pre-eclampsia (*n* = 17).

Pregnancy-induced hypertension was defined as two measurements of blood pressure obtained at different times (at least 4 h apart) over 140 mmHg systolic blood pressure or/and over 90 mmHg diastolic blood pressure with an appropriately sized cuff. American College of Obstetricians and Gynecologists criteria were further used for devising the hypertension group. [6] Pre-eclampsia was defined as a blood pressure measurement higher than 140 mmHg systolic blood pressure or/and above 90 mmHg diastolic blood pressure and a 24 h proteinuria of ≥ 300 mg/day. Severe pre-eclampsia was diagnosed based on a systolic blood pressure over 160 mmHg and/or 110 mmHg diastolic blood pressure from two 4 h-separate measurements and the presence of 24 h proteinuria over ≥ 500 mg/day, headaches or visual disturbances, upper abdominal pain, increased transaminases level of at least twice basal levels, creatinine over 1.1 mg/dL, thrombocytopenia under 100.000/µL, or pulmonary edema. 

Sociodemographic characteristics, obstetric history, family history, and clinical data were obtained from every patient using a standard interview. Venous blood samples for complete blood count, renal and hepatic function tests and urine samples for 24 h proteinuria were collected similarly for the entire study population as the standard prenatal assessment in our hospital. Blood samples for CA-125 estimation were also collected for every patient, and the samples were measured using the ECLIA method (electrochemiluminescent immunoassay). All included patients were followed up with until delivery, with records of delivery, maternal, and neonatal outcomes obtained after birth from the hospital database.

The Ethics Committee of the University of Medicine and Pharmacy “Victor Babeș” Timișoara provided ethical approval for the current study (No. 80/07.12.2020). Informed written consent was obtained from every pregnant woman enrolled in the study.

Statistical analysis was performed using Jamovi software (The Jamovi Project [2021]. Jamovi [Version 1.6] [Computer Software]. Retrieved from https://www.jamovi.org). (Accessed on 23.08.2022). Descriptive statistics were applied for all relevant data, and the normality of distribution was tested for all quantitative data. The t-test was used to compare the two groups and one-way ANOVA for the parametric variables of multiple groups associated with the Turkey post-test. Pearson’s correlation coefficient (Pearson’s r) was used to assess the correlation between different variables. A *p*-value of < 0.05 was considered significant.

## 3. Results

One hundred patients were enrolled in this study, and they were divided into four subgroups. The characteristics of the study population are shown in Table 1. The enrolled pregnant women ranged from 18–43 years old. The mean age for the normotensive group was 29.3 (±6.57), and the mean age for the hypertension group was 29.8 (±4.94). Parity ranged between 1 and 4 births. The mean gestational age at enrollment was 34.4 (±3.1), with no statistical difference between the two main groups of pregnant patients.

Regarding the study population’s medical history, 11% (*n* = 11) of the enrolled pregnant patients reported a history of pregnancy-related hypertension (any type), and 54% of those (*n* = 6) were from the hypertensive group. All patients from the hypertensive group were taking antihypertensive medication. Thirteen of the enrolled pregnant patients were taking low-dose aspirin for pre-eclampsia prophylaxis. Among those, 38,4% (*n* = 5) of patients were prescribed low-dose aspirin for a history of pregnancy-related hypertension, 7.6% (*n* = 1) of patients had an age-related and nulliparity as an indication for aspirin prophylaxis (over 40 years old), and 53.8% (*n* = 7) of patients were prescribed low-dose aspirin for non-hypertension related indications. Also, there was no statistical difference between the percentage of smokers in the normotensive group and in the hypertensive group.

The mean arterial pressure of the normotensive group was 90.4 mmHg (±9,9), while the hypertensive group had a mean MAP of 122.3 mmHg (±7.8). Significant differences between the MAP of normotensive patients and all the other subgroups were observed, as well as between MAP of patients with pregnancy-induced hypertension and patients with severe pre-eclampsia. The first-trimester uterine artery pulsatility index (UtPI) was available for 83% (*n* = 83) of the patients, and the second-trimester uterine artery pulsatility index was available for the remaining 17% (*n* = 17) patients. The clinical and biological parameters of each subgroup are shown in Table 2.

**Figure 1:**
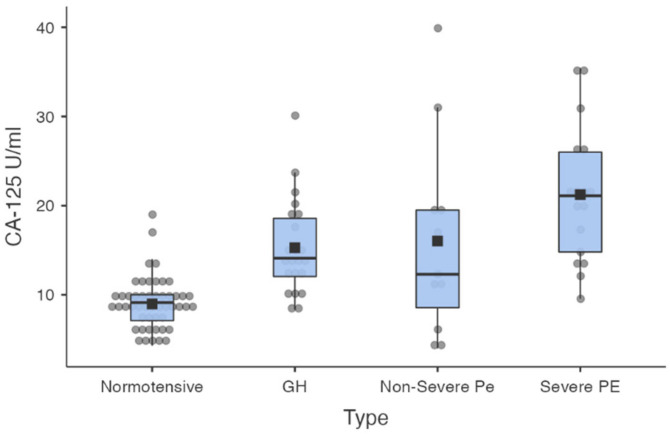
Mean values of CA-125 in the study population.

Mean CA-125 levels increased with the severity of pregnancy-induced hypertension from a mean of 8.97 U/mL (± 2.84) in the normotensive group to a mean of 21.23 U/mL (±11.18) in the severe pre-eclampsia group (Figure 1). Significant differences were observed between the normotensive subgroup and the hypertensive subgroups. Also, the severe pre-eclampsia group had a significantly higher mean level of CA-125 than the pregnancy-induced hypertension group and the non-severe pre-eclampsia group (Table 3).

There were no significant differences between subgroups regarding the mode of delivery due to the high rate of on-demand cesarean sections. However, significant differences were observed between the age of gestation at birth and the birth weight of the normotensive and pre-eclampsia groups. Also, a significant difference in the mean CA-125 levels was observed between newborns requiring NICU, 21.9 mUI/mL (±6.11), and newborns not requiring NICU, 16.1 (±4.01), *p*-value = 0.044. However, no correlation between CA-125 values and APGAR score was observed.

**Figure 2:**
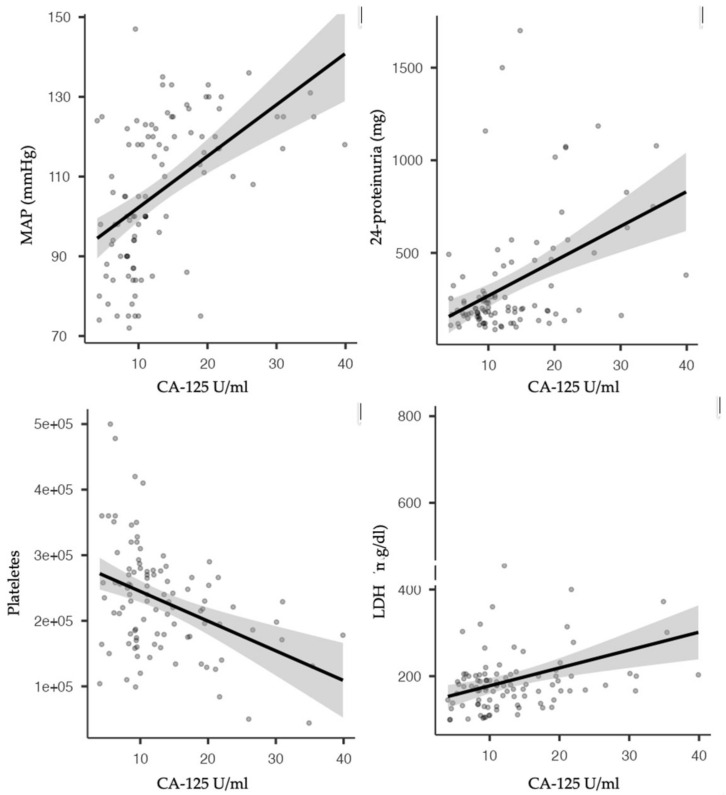
CA-125 correlation with MAP, 24 h proteinuria, platelets count, and LDH.

Several correlations were observed when analysing each parameter in relation to CA-125 levels. Mean arterial pressure and 24 h proteinuria were positively correlated with CA-125 values, while platelets count, gestational age at birth, and birth weight were negatively correlated with CA-125 (Figure 2.)

The other parameters, including first- and second-trimester uterine artery pulsatility indices, showed no relation (Table 4). Also, no significant mean CA-125 values were observed between women taking aspirin and those without aspirin.

**Figure 3:**
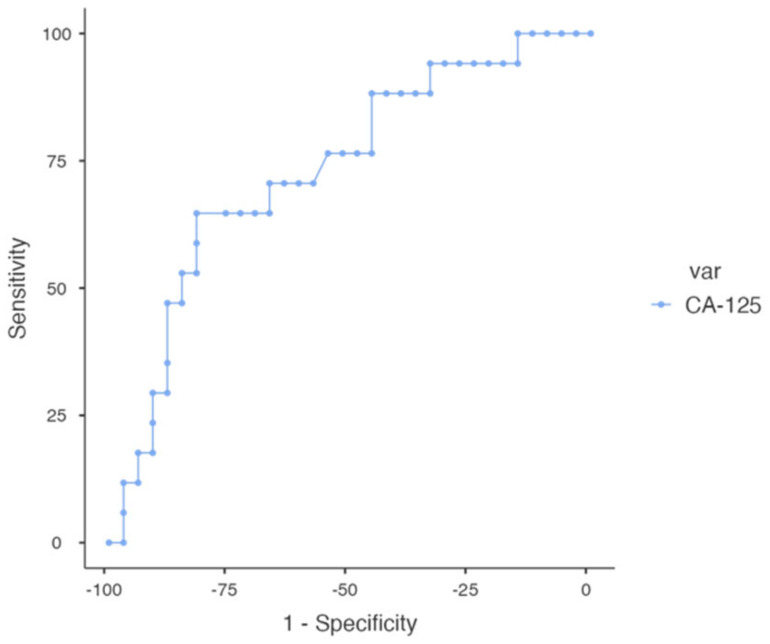
ROC curve of serum CA-125 and severe pre-eclampsia.

A cut-off value was calculated using the receiver operating characteristics (ROC) curve. The best value for severe pre-eclampsia, with a sensitivity of 64.71% and specificity of 81.82%, was a CA-125 of 19.8 U/mL. This level was associated with a negative predictive value (NPV) of 81.82% (Figure 3).

## 4. Discussion

Our study analysed the probability of a correlation between CA-125 levels and pre-eclampsia severity, thus allowing us to identify a possible severity marker that would improve the management of pre-eclampsia by selecting a high-risk group of patients for disease progression and aiding in the decision for hospitalisation and/or moment of birth.

The origin of CA-125 in patients with preeclampsia remains unclear, with some theories suggesting a relation with the presence of ascites or an increased expression due to the intense chronic inflammation process in the deficient placenta, as suggested by the increased CA-125 values associated with periods of physiological intense placental processes as in the first trimester or at birth. Moreover, correlation statistics from existing literature with other biochemical indices and fetal parameters offer additional evidence suggesting its contribution to the pathogenesis of the disease [24,25]. The use of CA-125 values in pre-eclampsia severity evaluation has been studied in a small number of papers, with some conflicting results. Up until the writing of this study, 16 studies published between 1993–2020 have been identified, involving a total number of 2453 pregnant patients, of which 1084 with hypertensive pregnancy pathology of various severities and 1369 healthy pregnant and non-pregnant patients in the control groups. Most studies have sub-grouped the study groups according to the severity of pre-eclampsia. The selected groups show considerable heterogeneity in terms of gestational age of inclusion. The definition of pre-eclampsia, and the forms of severity used to establish the study subgroups, were unanimous. The design of the studies was, except for 2 (cross-sectional) case–control studies.

Except for three studies, a significant correlation with the severity of pre-eclampsia was observed in all studies, similar to our results [26,27,28]. The common feature of these studies was the measurement of CA-125 after 20 weeks of gestational age, of which nine studies included pregnant women exclusively in the third trimester of pregnancy (>28 weeks/VG). Only pregnant women over 25 weeks of gestational age were enrolled in our study. All these studies reported significant differences between the mean value of CA-125 of the control group (healthy pregnant women) and the group with hypertensive pregnant women. Moreover, significant differences were observed with increasing severity of hypertension, particularly in the severe pre-eclampsia group. Our study observed lower mean values of CA-125 among subgroups, with a mean of 8.97 U/mL for the normotensive group compared with values ranging from 7.9 to 47.3 U/mL in published studies and a mean of 21.32 U/mL for severe pre-eclampsia compared with a range of 37.3 to 64.2 U/mL in other studies. Accordingly, the proposed cut-off in our study of 19.8 U/mL is also lower than the other proposed cut-offs of 23.7, 35, and 50 U/mL. However, cut-off values were reported in only three studies [29,30,31].

Correlations of CA-125 values with different clinical and biological parameters used in assessing the severity of pre-eclampsia have also been reported. Our study observed positive correlations with MAP, 24 h proteinuria, and LDH values and negative correlations with platelet count, gestational age at birth, and birth weight. Our findings are similar to most of the reported published data. No other published study has analysed CA-125 values in relation to ultrasound parameters with a known value in pre-eclampsia prediction [32]. In our research, the pulsatility index of the uterine artery measured in the first or second trimester was analysed, but unfortunately, no significant correlation was observed. Also, this is the first study reporting fetal outcomes in relation to CA-125 values, observing higher values in mothers whose newborns required NICU admission.

The observed statistical correlations with the standard biochemical parameters bring evidence that adding CA-125 measurements in clinical practice may offer an improved follow-up biochemical panel for pregnant patients with hypertension disorders aiding the clinician in the decision-making process regarding hospitalisation or outpatient management. As previously reported, some currently used biochemical parameters of hypertensive disorders, like proteinuria and uric acid, correlate poorly with unfavourable pregnancy outcomes [22,23]. The negative correlation with gestational age at birth and birth weight and the association of higher values of CA-125 with NICU admittance of the newborns may provide an additional factor in deciding the timing of delivery, as finding a balance between preventing worse maternal–fetal outcomes and possible complications of prematurity are essential. Our study observed higher CA-125 values (>30 U/mL) in pregnancies where birth had to be induced under 36 weeks of gestation. Similar observations were reported by Mukherjee and Geya [29,33].

In the only meta-analysis published on this subject, in 2019, which analyses nine studies identified up to that date, increased levels of CA-125 are reported in patients who develop preeclampsia in the third trimester, also showing significantly different values between control groups and preeclamptic pregnant women. The results obtained from the diagnostic accuracy analysis showed an AUC of 0.951, a sensitivity of the method of 91.9%, and a specificity of 86.9% for severe forms of preeclampsia.

Discrepancies between studies reporting the use of CA-125 in pre-eclampsia may be explained by the use of different estimation methods (ECLIA, ELISA, or RIA) and, most important, by the different biological and genetic characteristics of the population enrolled. This study is the first to involve the European population, as the other studies involved Asian and African populations.

## 5. Limitations

The heterogeneity and the small number of existing studies require caution in interpreting the results. This study provides additional data on a population not yet analysed in the literature. There were significant limitations in the sample size of the study group due to the hospital-based enrollment. Also, the inability to correlate CA-125 levels with the mode of birth is due to the current practices in our country.

## 6. Conclusions

The reported results support this marker’s promising role in managing pre-eclampsia as a severity marker. Further studies are needed in order to improve the accuracy of the obtained results, identify a high sensitivity and specificity cut-off, and achieve standardisation in measuring the marker. Moreover, to benefit from the predictive potential for pre-eclampsia severity, research focused on the optimal timing of the marker’s measurement is needed. Of course, an even more significant benefit would be its use as a first-trimester marker for pre-eclampsia. At the time of writing this article, no research on the dynamics and predictive value of CA-125 in the first trimester of pregnancy has been published.

## Figures and Tables

**Table 1:** Characteristics of the study population.

	Normotensive (*n* = 50)	Gestational Hypertension (*n* = 22)	Non-Severe Pre-eclampsia (*n* = 11)	Severe Pre-eclampsia (*n* = 17)	
Age	29.24 (±6.5)	29.05 (±4.8)	31.82 (±4.8)	29.65 (±4.9)	*p* = 0.45
GA at enrolment	33.96 (±3.1)	35.82 (±2.6)	34.55 (±2.9)	33.65 (±3.2)	*p =* 0.69
Parity	1.50 (±0.7)	1.36 (±0.7)	1.55 (±0.9)	1.40 (±0.7)	
GA at birth (weeks)	38.1 (±1.6)	37.1 (±2.22)	36.1 (±3.11)	34.9 (±3.53)	***p* = 0.003**
Birth weight (grams)	3194 (±405)	3295 (±552)	2695 (±905)	2343 (±899)	***p* = 0.005**

**Table 2:** Clinical and biological parameters of the study population.

	Normotensive (*n* = 50)	Gestational Hypertension (*n* = 22)	Non-Severe Pre-eclampsia (*n* = 11)	Severe Pre-eclampsia (*n* = 17)	
MAP	90.4 (±9.9)	119.8 (± 5.2)	119.5 (±6.02)	127.6 (±9.17)	***p* < 0.001**
UtPI T1	1.94 (±0.4)	1.89 (±0.4)	1.98 (±0.6)	2.45 (±0.6)	*p* = 0.144
UtPI T2	1.26 (±0.4)	1.18 (±0.5)	N/A	1.78 (±0.7)	*p* = 0.515
24-h Proteinuria (mg/24 h)	179 (±51.9)	177 (±51.9)	435 (±93.1)	897 (±366.7)	***p* < 0.001**
AST (mg/dL)	21.1 (±5.5)	18.6 (±3.7)	19.5 (±5.1)	49.5 (±57.9)	***p* = 0.046**
ALT (mg/dL)	22.1 (±5.8)	23.3 (±3.0)	23.8 (±5.29)	46.6 (±42.5)	*p* = 0.057
Creatinine (mg/dL)	0.79 (±0.1)	0.6 (±0.1)	0.73 (±0.1)	0.82 (±0.2)	*p* = 0.145
Platelets count	253,692/µL (±92,261)	241,727/µL (±44,121)	197,545/µL (±44,293)	167,000/µL (±73,610)	***p* < 0.001**
LDH (mg/dL)	171 (± 51.9)	158 (±26.7)	201 (±43.0)	287 (±157.9)	***p* = 0.020**
CA-125 (U/mL)	8.97 (± 2.84)	15.28 (±5.32)	16.06 (±11.18)	21.32 (± 7.64)	***p* < 0.001**

**Table 3:** Statistical significance of CA-125 levels between study subgroups.

Tukey Post-Hoc Test—CA-125 mUI/mL					
		GH	Non-Severe PE	Severe PE	Normotensive
GH	*p*-value	—	0.984	0.010	<0.001
Non-Severe PE	*p*-value		—	0.097	0.002
Severe PE	*p*-value			—	<0.001
Normotensive	*p*-value				—

**Table 4:** CA-125 levels’ correlations with clinical–biological parameters.

Parameter	Pearsons’s Correlation Coefficient (r)	*p*-Value
UtPI 1st T	0.097	0.190
UtPI 2nd T	0.189	0.226
MAP	0.510	* < 0.001
24-h proteinuria	0.441	* < 0.001
Platelets count	−0.403	* < 0.001
AST (mg/dL)	0.208	0.981
ALT (mg/dL)	0.204	0.979
Creatinine (mg/dL)	0.161	0.945
LDH	0.344	* < 0.001
Gestational age at birth	−0.259	* 0.005
Birth weight	−0.177	* 0.039

* MAP, mean arterial pressure; UtPI, uterine artery pulsatility index.

## Data Availability

Data are available from the first author upon reasonable request.
